# The Making of a Modern Hospital

**Published:** 1911-10-21

**Authors:** 


### THE MAKING OF A MODERN HOSPITAL.

### III.—Modern Hospital Systems.

The classification of modern hospitals outlined in the last article has shown that there is only one type of institution that may justifiably claim to be worthy of the title of a modern hospital in the sense we have already defined. This type is the general hospital. It, and it only, fulfils the functions of a hospital as distinct from those of an asylum: it alone, again, is able to bring into action against the forces of disease all the auxiliaries with which modern science has furnished modern medicine. In short, it is the type which may legitimately be regarded as representing a hospital at its best, and it is therefore the one which we have chosen to describe and discuss more particularly in this series.

### Hospital Systems.

Before doing so, however, it is necessary to say something with regard to the main principles on which hospitals exist, in other words briefly to consider the question of hospital systems. It is obviously impossible to deal with this subject, in all its bearings, within the limits of a single paper. All that can be done is to outline, in précis, the main points of the various existing systems of hospitalisation in the various countries. For further details regarding these we must refer our readers to the special articles which have already appeared and will appear again in The Hospital on the German system *: these articles are a first instalment of a series of papers dealing exhaustively with the hospital systems of the world, and in them our Special Commissioner has discussed the various points raised with some fulness and with instructive reference to the literature.

### The English System.

In England the general hospital is represented by two different types of institution, each managed on a totally different system. The large general hospitals in London and the provinces, the smaller and cottage hospitals, and the majority of the special hospitals, are supported under what is known as the Voluntary system: the Poor Law infirmaries, the fever hospitals, the naval and military hospitals, and a minority of special hospitals are supported by the tax-payers, either indirectly through State grants in maintenance or directly through the rates. England is therefore a "dual system" country so far as hospitals are concerned, but it differs from other countries where the dual system prevails in one important particular—namely, in the fact that medical education and the medical schools are associated wholly with institutions managed under the voluntary system. Some years ago there was an added distinction between the voluntary hospital and the Poor Law infirmary so far as this country was concerned. The latter was regarded with grave suspicion by the public, and patients only entered it as a last resource. Thanks to the modernisation of the large infirmaries and the careful selection of the staff by the Local Government Board, the infirmaries may now claim to be, in some cases at least, quite as good and as efficient institutions as the voluntary hospitals. Many of them receive private patients who pay special charges: in other cases again the superintendents have won a well-deserved reputation for their skill as consultants, while in a third group again special circumstances have combined to put the institution on a footing which no voluntary hospital can rival. The two largest children's hospitals in this country are institutions under the Local Government Board, and the excellence, so far as diagnosis and treatment are concerned, of the fever hospitals is well known.

### The Question of Control.

The institutions managed under the voluntary system are generally financed and controlled by the charitable public on thoroughly democratic lines. A discussion of the many side issues and the pros and cons of the system would be out of place here, but readers of this journal need not be told of the striking success which has attended the working of the voluntary system in this country. Notwithstanding this success, however, it is a remarkable fact that in no other country is the system honoured to such an extent as in England. The dual system, sometimes considerably modified, is found all over the civilised world, but wherever it is found, except in England, there exist arrangements whereby the State, as such, exercises a certain degree of control and supervision over all private institutions for the sick. In Turkey, for example, where there are at the present time several interesting hospitals managed under the voluntary principle, the State reserves to itself the right not only of inspecting the buildings, but also of supervising the expenditure of the money which has been contributed towards the endowment funds. In Roumania the State exercises a similar right, while in other countries it delegates certain supervisory powers to local authorities. A comparison of the methods of establishing a general hospital in this country and in other countries where the dual principle obtains may be interesting, therefore, in showing the differences that exist.

### Local Differences.

With us anyone, whether individual or corporation, can establish or found a hospital. A rich man, willing to expend half a million pounds on a hospital as a hobby, may build, equip, staff, and run a large general hospital in the heart of London, and no one can interfere with his institution so long as it is run on rational lines or in such a manner as not to give rise to a public scandal. It may appeal for subscriptions, establish a medical school, make what rules and regulations it likes, and to a certain extent it occupies a privileged position. Ten years ago a patient badly treated in such an institution would probably have had no chance of obtaining damages against the management. Even to-day, when the American courts have definitely laid it

* "Hospitals and State Aid." The Hospital, June 17, 1911, pp 295-297; July 1, p. 375. "The German System." The Hospital, June 24, July 1, July 8, July 15.

down as a rule—and one which it is very likely will now be recognised by our own courts—that a charitable institution is liable for damages just as much as an ordinary practitioner is, there is some doubt as to whether such damages will not be merely nominal when it can be shown that the hospital was a bona-fide charitable institution. As we shall show in the following articles, the responsibilities of hospital founders and boards of management are very heavy: the pity of it is that they are so often lightly regarded and that under our system, benevolent, well-intentioned, but ill-advised individuals or corporations build hospitals without first of all carefully considering their obligations to the public, existing hospitals, and their own institution. This disadvantage attached to our system has long been felt by hospital workers, and earnest attempts have been made to find some means whereby an effective control, in the interests of the public, can be exercised over our voluntary hospitals without at the same time interfering unduly with their independence or curtailing in any way the working of the voluntary system. The King's Hospital Fund, the Saturday and Sunday Hospital Funds, and to a much slighter extent the Central Hospitals Council, provide, within certain limits, such control. By withholding grants to hospitals which are badly managed or mismanaged the King's Fund, for instance, is in a position to exercise some control over such institutions. It is true it has no power to enforce compliance with its recommendations, but the mere fact of withholding a grant, by reason of its drawing public attention to the faults of management in the institution in question, serves very well to maintain the high standard of efficiency and thoroughness which it is in the interests both of the hospitals and the public to insist upon for all voluntary institutions.

### The State Aid System.

In a country where voluntary hospitals exist in the minority, the majority of institutions being under State or municipal control, no one can found such a hospital without first of all obtaining the approval of the State or local authorities. The plans for the new institution must be submitted to these authorities for the inspection and approval of experts; the regulations drawn up must be approved, and must, in any case, be in accordance with certain provisions specifically drawn up by the legislature. In such countries, too, all voluntary hospitals are liable to be inspected by Government or municipal inspectors, who may not only report and recommend alterations, but have the power to enforce compliance with their recommendations. As a set-off against these restrictions the institutions are granted certain privileges, such as the right to hold land, to have members of their staff recognisd as professors or teachers, exemption from taxation, or special favours which vary in importance in proportion to the size and usefulness of the institution. These privileges, it is considered, are necessary to enable the institutions to compete with the well-supported State or municipal hospitals which form the large majority of institutions for the sick in other countries. It is recognised, for example, that there may be special reasons which influence a section of the community to found its own hospital. One of these reasons is the difference in religious customs which has led to the foundation, in Christian communities, of Jewish hospitals, such as exist at Berlin, or of Catholic hospitals in Protestant districts, as in north Germany. Generally, in countries where the voluntary system does not prevail to the same extent as it does in England, the State and the local authorities share the main burden of the upkeep of the hospitals. In every country it is the State that controls, and provides for, the special State hospitals attached to the army and navy, the prisons, and reformatories, and State colonies and epidemic hospitals. In some places the State also controls the majority of large general hospitals. This is notably the case in Austria, where municipal hospitals are a comparatively recent innovation—Vienna, for example, has only got one municipal hospital, which was founded within the last four years—and in our own Crown colonies (see "Hospitals and State Aid," The Hospital, July 1).

As a rule, however, the large modern general hospitals are under municipal control in most civilised countries, with the exception of England. Such control may be exercised directly, as in Germany and the United States, or indirectly, as in Italy; or, again, it may be exercised in combination with a special department of State, as in France. The main fact is that provision is made for control to the detriment of the voluntary principle as such.

It is evident from the foregoing brief summary of comparative conditions that we have to divide our general hospitals into two large classes—voluntary hospitals, democratically managed, and State and municipal aided institutions managed by a bureaucracy. This distinction it is worth bearing in mind since the two systems have had an important bearing on the making of a modern hospital, as we shall endeavour to explain in future articles. It has been necessary to deal in these preliminary papers with general considerations so as to clear the ground for a consideration of the details of hospital construction and administration, and it is now possible to proceed with the latter. In the next article we will therefore deal with what constitutes a modern hospital in the fullest sense of the term.

---

Note.—Our attention has been drawn to the fact that the definition of a general hospital as given in the last article is not the one usually accepted, since it is commonly held that the term "general" applies not to the conditions from which patients suffer but to the area from which the institution draws its patients. Thus a special hospital, devoted only to the treatment of one class of case (as the Bath hospital and some eye hospitals), may arrogate to itself the title of a general hospital on the assumption that it receives patients not merely from its immediate population but from everywhere. While we recognise this distinction we hold, in agreement with most modern authorities, that this use of the term "general" is no longer justifiable, and that the definition we have given is in the circumstances a fair one for a modern general hospital.—Ed., The Hospital.

